# Supplementary material for: CNA Profiling of Single CTCs in Locally Advanced Esophageal Cancer Patients during Therapy Highlights Unexplored Molecular Pathways
Source: Cancers (Basel). 2021 Dec 19;13(24):6369. doi: 10.3390/cancers13246369 (PMC8699413; doi:10.3390/cancers13246369)
Supplement: Supplementary file 1 [file cancers-13-06369-s001.zip › cancers-1497703-supplementary/cancers-1497703-suppl-proof/cancers-1497703-suppl-layout.pdf]

cancers

MDPI

Supplementary Materials

CNA Profiling of Single CTCs in Locally Advanced Esophageal Cancer Patients during Therapy Highlights Unexplored Molecular Pathways

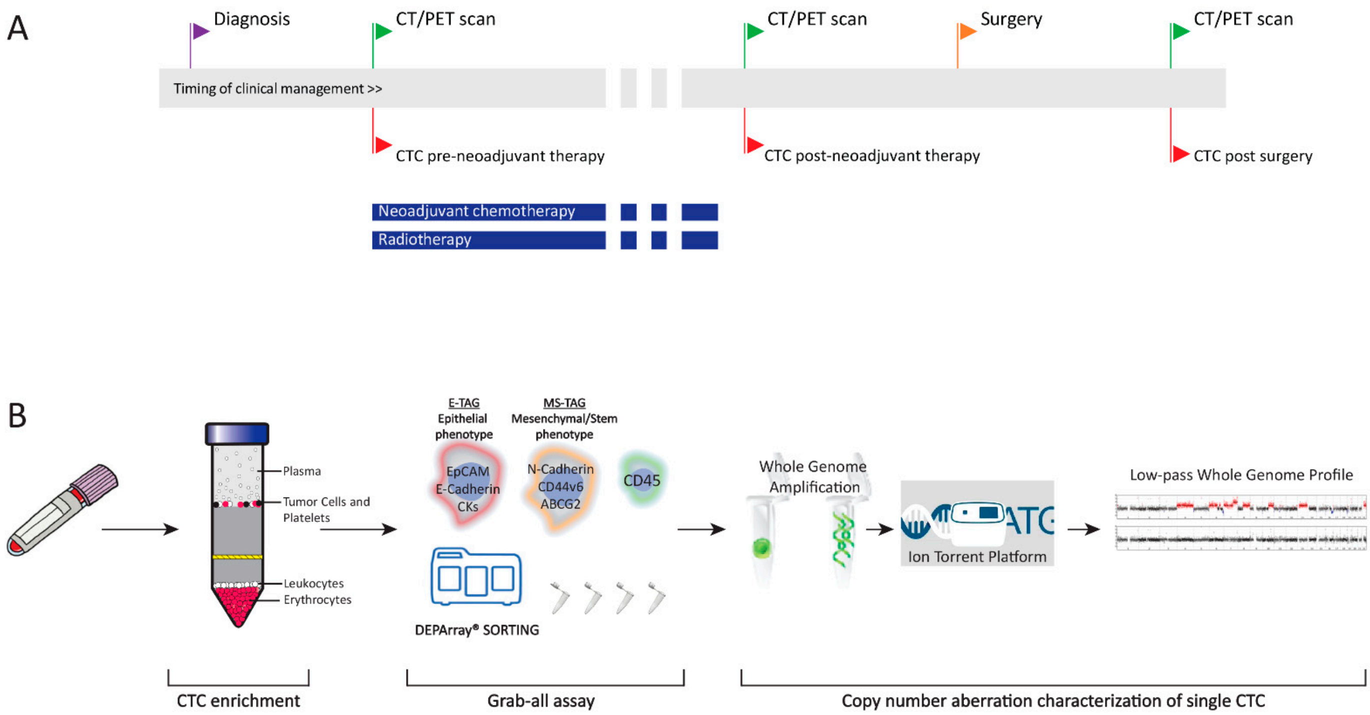

Figure S1. Clinical history of esophageal cancer (EC) patients and Experimental Workflow. (**A**) Outline of the clinical therapeutic history of patients with non-metastatic EC. Along the timeline, patient's clinical management milestones are paired with blood biopsies (red flags). Treatment regimens are shown as blue bars below the timeline. (**B**) Experimental workflow adopted for CTC analysis. CTC enrichment from peripheral blood was performed by density gradient based Oncoquick, followed by Grab-all assay to identify CTCs with different phenotypes using DEPArray platform. Once identified, CTCs were sorted as single cells and subjected to whole genome amplification. Low-pass whole genome profiles were obtained from amplified DNA of single CTCs using the Ion Torrent S5 platform. CTC: circulating tumor cell; CT/PET scan: Computed Tomography/Positron Emission Tomography scan.

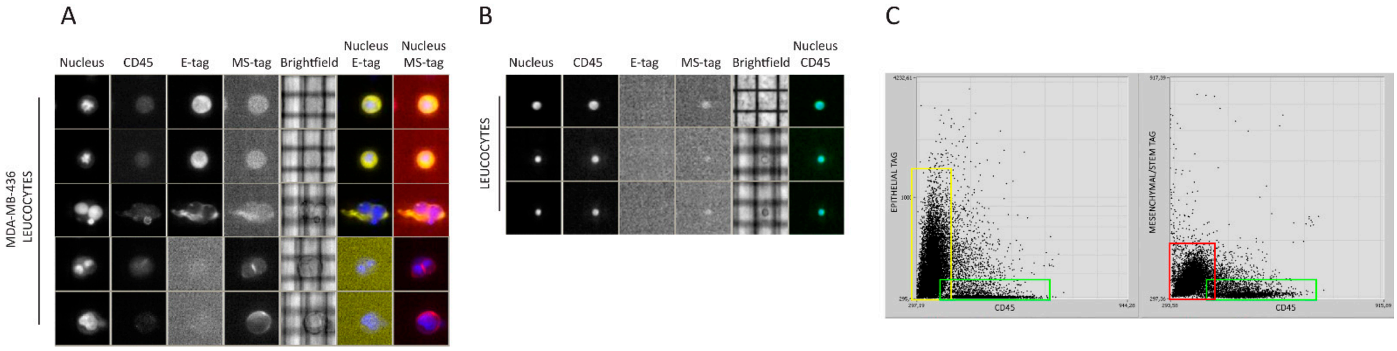

Figure S2. Validation of the Grab-all assay by DEPArray acquisition. (**A**) DEPArray panel of MDA-MB-436 cells stained with CD45 and E-tag antibodies, CD45 and MS-tag antibodies. Time exposures of every fluorescence channel were defined

separately to avoid misinterpretations. (**B**) The Grab-all assay was tested on leukocytes to measure non-specific signals as well as to complete the acquisition setup. (**C**) Representative DEPArray dotplot graphs of a mixture of MDA-MB-436 cells and leukocytes. On the left, the dotplot graph of the epitelial tag mean intensity (y-axis) and CD45 mean intensity (x-axis). Yellow and red squares identify MDA-MB-436 cells stained with epithelial and mesenchymal/stem tags, respectively. Leukocytes are gated on green squares. E-tag: epithelial phenotype tag; MS-tag: mesenchymal-stem phenotype tag.

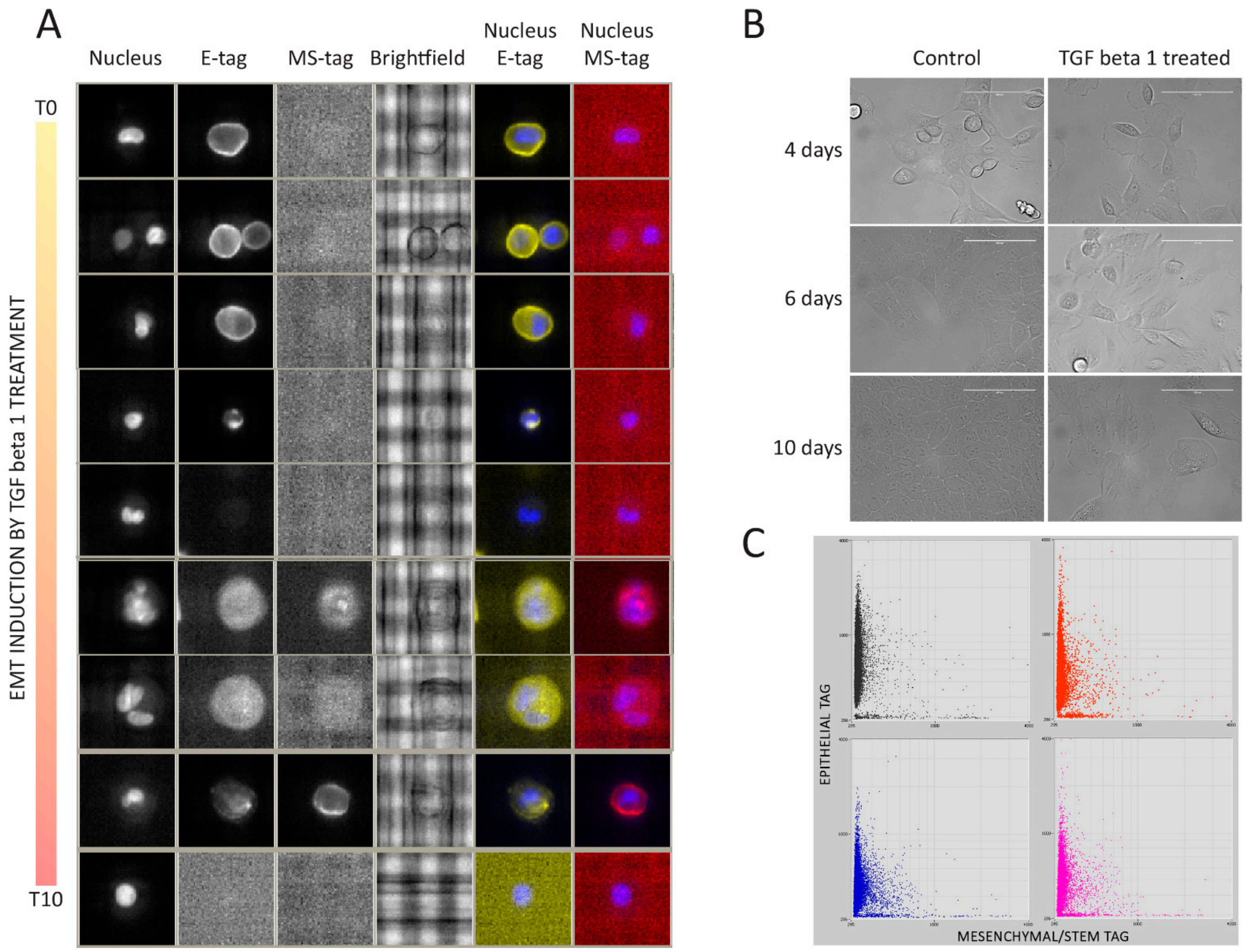

Figure S3. Validation of the Grab-all assay on MCF10A cells induced in EMT using TGF beta-1 treatment. (**A**) DEPArray panel of EMT-induced MCF10A. Representative images of single MCF10A without TGFbeta-1 treatment (T0) and during ten days of treatment (from the top to the bottom). Cells were stained with the Grab-all assay, which clearly detects the EMT-associated phenotypic change from a homogeneously frankly epithelial MCF10A to a more mesenchymal population. Cell morphology of MCF10A cells treated with TGF beta-1 during EMT (**B**). EMT: epithelial to mesenchymal transition; TGF beta-1: Transforming growth factor beta 1.

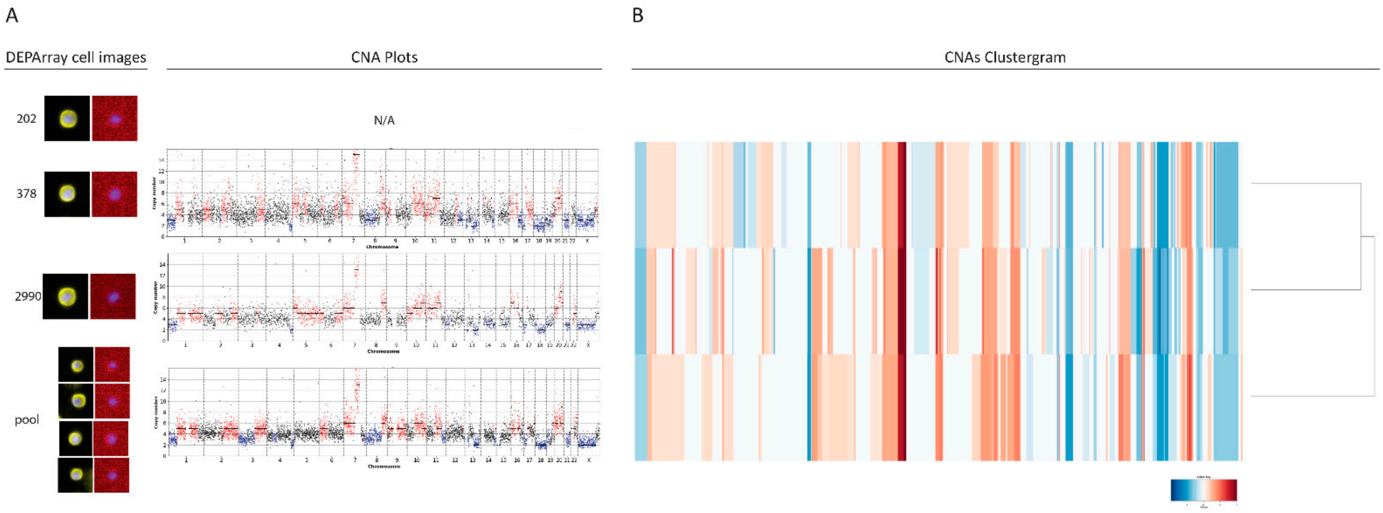

Figure S4. Example of copy number profiles of single and pooled OE-33 cell line with matched DEPArray images (**A**). Clustering analyses of the same samples (**B**) revealing high CNA concordance between single and pooled cells. CNA: copy number aberration; N/A: not applicable.

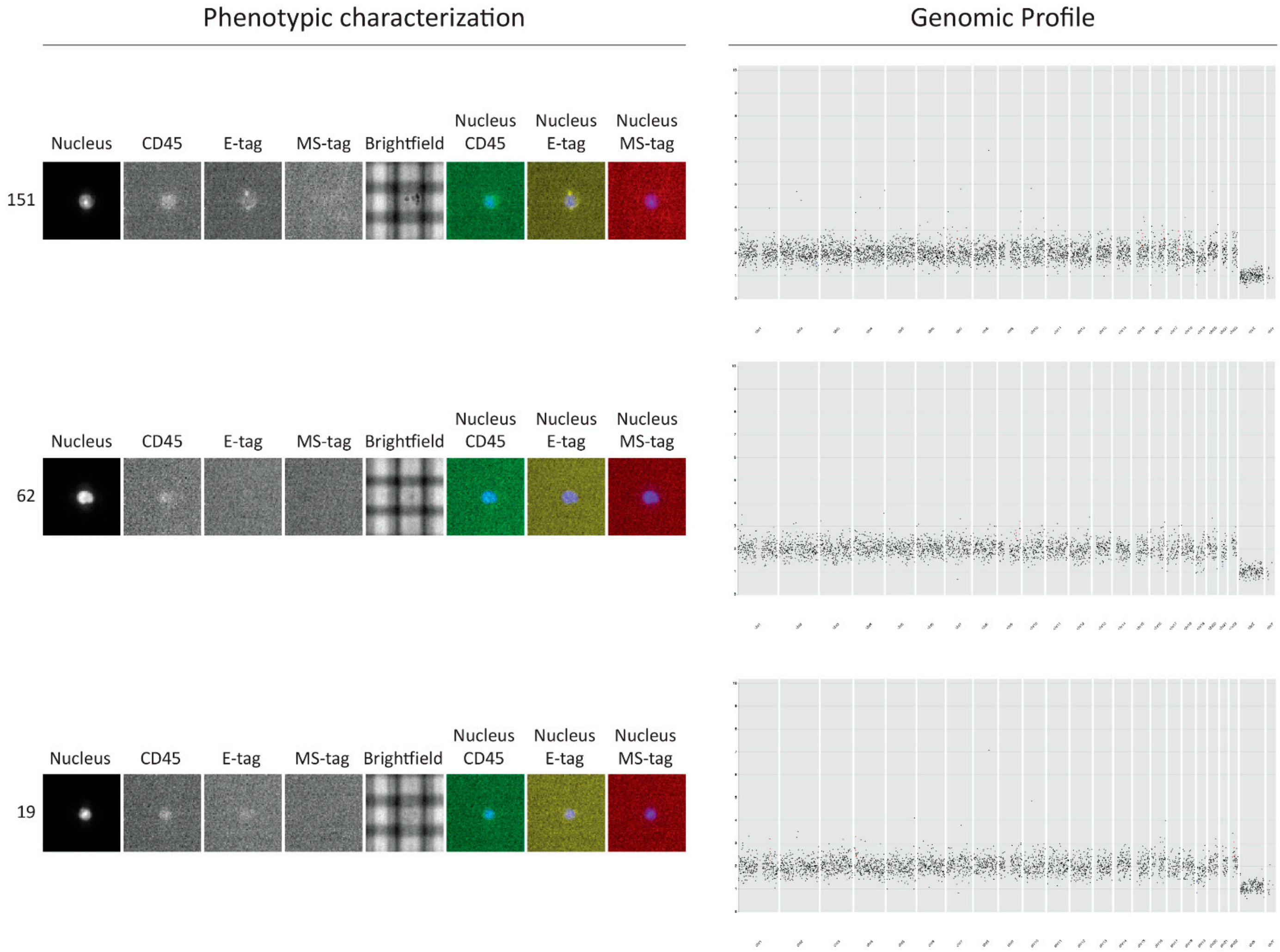

Figure S5. Analysis of undefined cells obtained from peripheral blood of patient NM8 after neoadjuvant therapy. On the left representative DEPArray images of NM8 cells that were negative for both CD45 and phenotypic tags. Single cells were sorted and sequenced. Their CNA profile revealed a normal diploid chromosomal set. Therefore patient NM8's CTC status

after neoadjuvant treatment was assigned as negative. CNA: copy number aberration; E-tag: epithelial phenotype tag; MS-tag: mesenchymal-stem phenotype tag.

Table S1. Clinical-pathological characteristics of metastatic esophageal cancer blood donors and phenotypic characterization of identified CTCs. Histological type: EAC esophageal adenocarcinoma and ESCC esophageal squamous carcinoma. M: male; CTC: circulating tumor cell.

Patient	Sex	Age	Histological Type	Tumor Grade	Metastatic sites	First line chemotherapy	Total Epithelial CTCs	Total Mesenchymal/stem CTCs
**M1**	M	71	ESCC	G3	Liver	5-Fluorouracil	1	24
**M2**	M	41	EAC	G3	Liver, lymph nodes, bones, lung, peritoneum	FOLFOX	2	0
**M3**	M	43	EAC	G3	lymph nodes, brain, bones	FOLFOX	5	2
**M4**	M	73	EAC	G3	Liver	Cisplatin/ 5-Fluorouracil	7	0

Table S2. Clinical characteristics of enrolled patients affected with non-metastatic esophageal cancer (EC). Histological type: EAC esophageal adenocarcinoma and ESCC esophageal squamous carcinoma. Disease stage at diagnosis as clinical classification (cTNM). Pathological classification (ypTNM) is shown for the patients who underwent surgery. Only patients defined as «stable disease» or responding to therapy by CT/PET scan were eligible for surgery. Post therapy CT scan defined as partial response (PR), stable disease (SD) or not evaluated (NE). N/A as not available. CT/PET scan: Computed Tomography/Positron Emission Tomography scan.

Patient	Sex	Age	Histological Type	Tumor Grade	Disease stage at diagnosis	Neoadjuvant chemotherapy	Concomitant Radiotherapy	Post-therapy CT	Surgical resection	ypT Stage
**NM1**	F	62	EAC	G2	cTxN2M0	Cisplatin/ 5-Fluorouracil	48.60 Gy in 27 fractions	PR	No	N/A
**NM2**	M	72	EAC	N/A	cT3N2M0	Cisplatin/ 5-Fluorouracil	50 Gy in 25 fractions	PR	Yes	T0N0M0
**NM3**	M	71	EAC	G3	cT2N2M0	Carbotaxol	41.4Gy in 23 fractions	NE	No	N/A
**NM4**	M	62	EAC	G3	cT4bN2M0	Epirubicin/ Oxaliplatin/ 5-Fluorouracil	50Gy in 25 fractions	SD	Yes	T3N0M0
**NM5**	M	73	EAC	G2	cT3N1M0	Carbotaxol	45 Gy in 25 fractions	SD	Yes	T0N0M0
**NM6**	M	45	ESCC	N/A	N/A	Cisplatin/ 5-Fluorouracil	45 Gy in 25 fractions	PR	Yes	T3NxM0
**NM7**	F	54	ESCC	G2	cTxN2M0	Carbotaxol	45 Gy in 25 fractions	PR	No	N/A
**NM8**	M	51	EAC	G3	cT4aN3M0	Cisplatin/ 5-Fluorouracil	50.4 Gy in 28 fractions	PR	Yes	T3N0M0
**NM9**	F	55	EAC	G3	cT3N2M0	Cisplatin/ 5-Fluorouracil	50.4 Gy in 28 fractions	PR	Yes	T1N1M0
**NM10**	M	76	EAC	G3	cT3N1M0	Carbotaxol	50 Gy in 25 fractions	PR	Yes	T3N1M0
**NM16**	M	60	ESCC	G2	cT3N2/3M0	Carbotaxol	50.4 Gy in 28 fractions	PR	Yes	T2N3M0

Table S3. List of enriched terms of all single CTCs according to Gene Ontology biological process. CTC: circulating tumor cell.
